# Immune proteomic associations with indices of cortical development in 5‐year‐old children

**DOI:** 10.1111/joa.70225

**Published:** 2026-09-10

**Authors:** Aaron Barron, Aylin Rosberg, Jetro J. Tuulari, Minna Lukkarinen, Harri Merisaari, Eero Silver, Venla Kumpulainen, Anni Copeland, Ekaterina Saukko, Alex M. Dickens, Matej Oresic, Tuulia Hyötylainen, Linnea Karlsson, Hasse Karlsson, Isabella L. C. Mariani Wigley, Elmo P. Pulli

**Affiliations:** ^1^ FinnBrain Birth Cohort Study, Turku Brain and Mind Centre, Department of Clinical Medicine University of Turku and Turku University Hospital Turku Finland; ^2^ Centre for Population Health Research Turku University Hospital and University of Turku Turku Finland; ^3^ Department of Psychiatry Turku University Hospital and University of Turku Turku Finland; ^4^ Clinical Neurosciences University of Turku Turku Finland; ^5^ Neurocenter Turku University Hospital Turku Finland; ^6^ Department of Paediatrics and Adolescent Medicine Turku University Hospital and University of Turku Turku Finland; ^7^ Department of Diagnostic Radiology University of Turku Turku Finland; ^8^ Department of Radiology Turku University Hospital and University of Turku Turku Finland; ^9^ Turku Bioscience Centre University of Turku and Åbo Akademi Turku Finland; ^10^ Department of Chemistry University of Turku Turku Finland; ^11^ School of Medical Sciences Örebro University Örebro Sweden; ^12^ School of Science and Technology Örebro University Örebro Sweden; ^13^ Department of Child Psychiatry Turku University Hospital and University of Turku Turku Finland; ^14^ Department of Clinical Medicine, Unit of Public Health University of Turku and Turku University Hospital Turku Finland

**Keywords:** child, growth, immune activation, immune proteomics, inflammation, MRI

## Abstract

The immune system has emerged as a critical factor influencing neurodevelopment in offspring. While this is increasingly well‐studied in the context of maternal immune activation (MIA), the relationship between postnatal immune activation and brain development in childhood is far less studied. In the current study, we performed targeted immune proteomics to analyse 356 inflammation‐associated proteins and examine whether peripheral immune physiology is associated with multimodal neuroimaging metrics, brain growth centiles, and cortical microstructure, in 5‐year‐old children. We included 101 typically developing children with immune proteomics and multimodal neuroimaging data covering structural and diffusion‐weighted magnetic resonance imaging (MRI) that was modelled with FSL's linked independent component analysis (FLICA) to yield 10 multimodal brain components. For a subset of the analyses utilising immune proteomics and structural brain growth centile data, the sample size was 126 children. We observed consistent negative associations between serum immune proteins and both grey matter volume growth and global cortical thickness; for the latter, associations were statistically significant after false discovery rate correction for CXCL10, IL17A, and LILRB4. Similarly, many immune proteins were associated with increased mean diffusivity across the cortex, most notably LAMP3, NCR1, SIGLEC1, FASLG, and CXCL10 in the right medial orbitofrontal cortex. Collectively, these findings provide the first characterisation of the relationship between immune physiology and brain structure in children, and demonstrate notable associations with global cerebrocortical development. This should be extended by future studies with larger samples and multiple sampling.

## Graphical abstract

Typically developing 5‐year‐old children (*n* = 101–126) from the FinnBrain Birth Cohort Study, Finland, underwent both targeted serum immune proteomics and magnetic resonance brain imaging. Multiple inflammatory markers, from one cluster of co‐expressed immune proteins, were negatively associated with indices of cortical development, including thickness, volume, and mean diffusivity.

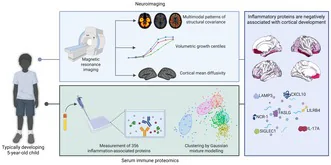

## 1 INTRODUCTION

The immune system plays a crucial role in brain development, shaping both early neurodevelopmental processes and later structural and functional outcomes. Maternal immune activation (MIA) has been linked to increased risk for neurodevelopmental disorders such as autism spectrum disorder, with animal models implicating mechanisms such as microglial activation, altered cytokine signalling, and disrupted synaptic pruning and neurogenesis (Boulanger‐Bertolus et al., 2018; Jones & Van de Water, 2019; Lin & Chen, 2022; Mastenbroek et al., 2024; Prins et al., 2018). Recently, human neuroimaging studies have begun to reveal associations between gestational inflammatory markers, such as C‐reactive protein (CRP) and interleukin‐6 (IL‐6), and alterations in brain structure and connectivity, including changes in the salience network, amygdala, cerebellum, and cortical morphology in neonates (Graham et al., 2018; Niskanen et al., 2025; Spann et al., 2018; Suleri et al., 2022). While the influence of maternal immune activation during pregnancy on offspring neurodevelopment has been extensively studied in recent years, the impact of postnatal immune processes remains underexplored, especially in humans.

The role of immune signalling in shaping human brain development is most likely not refined to the prenatal period, but continues to influence key neurodevelopmental processes postnatally, including but not limited to synaptogenesis, programmed cell death, myelination, and the refinement of neuronal circuits (Boulanger, 2009; Budday et al., 2015; Crampton et al., 2012; Cunningham et al., 2013; Kummer et al., 2021; Menassa & Gomez‐Nicola, 2018; Morimoto & Nakajima, 2019). Processes such as synaptogenesis and myelination peak during early childhood (Bethlehem et al., 2022; Zeiss, 2021), a period marked by rapid grey and white matter growth and heightened sensitivity to environmental and biological influences. Some preclinical and neonatal clinical neuroimaging studies have examined early postnatal infection in relation to neurodevelopment, revealing adverse neurodevelopmental outcomes such as reduced hippocampal volume and impaired hippocampal function in mice (Malaeb et al., 2014) and decreased white matter fractional anisotropy (FA) in human neonates (Chau et al., 2012; Glass et al., 2018). To the best of our knowledge, no neuroimaging study has yet investigated the relationship between inflammatory biomarkers and any brain structural parameters in early childhood. Most of the available evidence is limited to cognitive measures, for instance, negative associations between inflammation markers such as IL‐6 and cognitive school readiness in preschool‐aged children (Sutter et al., 2018) or academic success in children and adolescents aged 7–14 years (Esteban‐Cornejo et al., 2016). This lack of comprehensive neuroimaging studies addressing early childhood inflammation and neurodevelopment indicates a significant gap in this area of research.

The biological mechanisms by which inflammation affects brain development have been examined in rodent studies. IL‐6 and interleukin 17A (IL‐17A) in particular, two inflammatory cytokines which coregulate each other's expression, have been shown to be responsible for the neurodevelopmental effects of MIA on the offspring brain and behaviour in animal models (Choi et al., 2016; Kummer et al., 2021; Wu et al., 2017), although it is unknown whether this cytokine specificity is conserved in humans. While some studies have reported an association between maternal IL‐6 and offspring brain development up to 2 years (Graham et al., 2018; Rasmussen et al., 2019; Spann et al., 2018), this has not yet been explored in the context of postnatal cytokine levels during childhood. Notably, most pre‐existing studies examine only one or a small number of cytokines, and thus it is unknown whether there are in fact other immune biomarkers more closely related to brain development that remain unexplored.

Thus, the role of postnatal immune activation in shaping brain development in young children remains poorly understood, despite the fact that brain maturation continues dynamically in the years after birth. The current study aims to address this question by combining comprehensive serum immune proteomics with brain growth and multimodal neuroimaging data in early childhood. We explore the associations between 356 inflammation‐associated proteins and grey and white matter development through brain growth centiles and data‐driven structural components in 5‐year‐old children from a general population‐based cohort study. This study is exploratory with regard to brain metrics used, as they are novel to the field, and regarding the immunological markers as we sought to establish associations across the entire immune proteome rather than a small number of cytokines.

## 2 METHODS

This study was conducted in accordance with the Declaration of Helsinki, and it was approved by the Joint Ethics Committee of the University of Turku and the Hospital District of Southwest Finland (VARHA/18,203/13.02.02/2023).

### 2.1 Participants

The participants are a part of the FinnBrain Birth Cohort Study (www.finnbrain.fi). For cohort information, see Karlsson et al. (2018). The present study included children with blood samples and neuroimaging data at the age of 5 years. Blood samples were collected as part of the paediatric study visits (mean age 5.57, SD 0.11 years, for the sample used in this study). Study nurses contacted around 1200 families, of whom 941 children attended the 5‐year paediatric study visit. Serum samples were successfully collected from 807 children during this visit. Serum immune proteomics was analysed for 779 participants, and 776 (99.6%) of those were successful. Neuroimaging visits were attended by 203 participants (113 boys (55.7%), mean age 5.40 (SD 0.13), range 5.08–5.79 years). None of the children in this study had acute illness or infection at the time of sample collection, and all had CRP within a normal physiological range (median 0.2 mg/L, range < 0.01–3.52 mg/L).

For this study, only participants with both an adequate quality structural T1‐weighted image (*n* = 173/203, assessed by E.P.P. as described in (Pulli et al., 2022)) in combination with good‐quality diffusion tensor imaging (DTI) data (*n* = 147, assessed by V.K., see Kumpulainen et al. (2022)) and successfully processed immune protein measurements were included. The overlap between structural images and immune protein measurements (used for growth chart analysis) was 126. The overlap between all three datasets (used for FLICA analysis) was 101. The demographic information is presented in Table 1.

**TABLE 1:** Demographic information of the participants for the growth chart analysis sample and FLICA sample separately.

Continuous variables	Growth chart (*n* = 126)		FLICA (*n* = 101)	
	Mean (SD)	Range	Mean (SD)	Range
Age at scan (years)	5.39 (0.13)	5.08–5.79	5.37 (0.11)	5.08–5.79
Age at blood sample (years)	5.57 (0.12)	5.29–5.90	5.55 (0.11)	5.29–5.90
Gestational age at birth (weeks)	39.7 (1.6)	33.9–42.1	39.8 (1.5)	33.9–42.1
Ponderal index at scan	13.9 (1.1)	11.2–17.6	13.9 (1.2)	11.2–17.6
Maternal BMI before pregnancy	23.5 (4.0)	17.5–42.0	23.3 (3.4)	17.5–33.8
Maternal age at term (years)	31.2 (4.6)	21.5–42.0	31.1 (4.5)	21.5–42.0

Categorical variables	Growth chart	FLICA
	Number (%)	Number (%)
*Sex*		
Male	69 (54.8)	51 (50.5)
Female	57 (45.2)	50 (49.5)
*Maternal education level*		
Low	26 (20.6)	21 (20.8)
Middle	35 (27.8)	28 (27.7)
High	65 (51.6)	52 (51.5)
*Paternal education level*		
Low	26 (20.6)	20 (19.8)
Middle	32 (25.4)	27 (26.7)
High	31 (24.6)	27 (26.7)
Missing	37 (29.4)	27 (26.7)
*Maternal monthly income, estimated after taxes (euros)*		
≤1500	42 (33.3)	28 (27.7)
1501–2500	66 (52.4)	57 (56.4)
2501–3500	12 (9.5)	10 (9.9)
≥3501	1 (0.8)	1 (1.0)
Missing	5 (4.0)	5 (5.0)
*Maternal background*		
Finnish	117 (92.9)	93 (92.1)
Other or mixed	5 (4.0)	4 (4.0)
Missing	4 (3.2)	4 (4.0)
*Gestational age at birth*		
Term, gestational weeks ≥37	118 (93.7)	96 (95.0)
Preterm, gestational weeks <37	8 (6.3)	5 (5.0)
*Stay in neonatal intensive care unit*		
Yes	17 (13.5)	13 (12.9)
No	109 (86.5)	88 (87.1)
*Alcohol use during pregnancy*		
Yes, continued to some degree after learning about pregnancy	19 (15.1)	15 (14.9)
Yes, stopped after learning about pregnancy	15 (11.9)	12 (11.9)
No	91 (72.2)	73 (72.3)
Missing	1 (0.8)	1 (1.0)
*Tobacco smoking during pregnancy*		
Yes	7 (5.6)	4 (4.0)
No	119 (94.4)	97 (96.0)
*Illicit drug use during pregnancy*		
No	119 (94.4)	94 (93.1)
Missing	7 (5.6)	7 (6.9)
*Pregnancy complication*		
Yes	20 (15.9)	16 (15.8)
No	106 (84.1)	85 (84.2)

*Note*: Ponderal index was calculated using the following formula: weight in kilograms divided by height in metres cubed. Height and weight were acquired during the neuroimaging visit. The participants kept indoor clothes on during the weighing. Ponderal index information was missing for one of the growth chart sample participants. Maternal and paternal education data were combined from questionnaire data from 14 weeks gestation or 5 years of child age by choosing the highest degree reported. The three classes are: Low = Upper secondary school or vocational school or lower, Middle = University of applied sciences, High = University. The data for maternal monthly income estimate and illicit drug use are from questionnaires at gestational Week 14. On the question about alcohol usage, four subjects answered that they did not use alcohol during pregnancy but also answered that they stopped using alcohol when they learned about the pregnancy. These were classified as ‘yes, stopped when learning about pregnancy.’ The pregnancy complications include a diagnosis (according to ICD‐10) for O12 (Gestational oedema and proteinuria without hypertension), O13 (Gestational hypertension without significant proteinuria), O14 (Severe pre‐eclampsia), O24 (Diabetes mellitus in pregnancy, childbirth, and the puerperium), O46 (Antepartum haemorrhage, not elsewhere classified), or O99.0 (Anaemia complicating pregnancy, childbirth and the puerperium). Sex, birth weight, maternal BMI before pregnancy, and smoking data (combined with questionnaire data) were retrieved from the well‐being services county of Southwest Finland (VARHA).

Abbreviations: BMI, body mass index; FLICA, FSL's linked independent component analysis; GW, gestational week; SD, standard deviation.

### 2.2 Serum immune proteomics

A blood sample was collected at the 5‐year paediatric study visit, and serum fraction was separated from whole blood, aliquoted, and stored at –80°C. Immune proteomics was analysed using the Olink® Explore Inflammation 384 panel, a proximity‐based assay where specific oligonucleotides bound to targeted antibodies hybridise upon antibody binding and are then detected by next‐generation sequencing (https://olink.com/products/olink‐explore‐3072‐384). Normalised protein expression was calculated from the next‐generation sequencing read counts. Complete data were generated for 356 inflammation‐associated proteins in 776 children, and the current study used the normalised expression of all proteins for the subset of children who also had neuroimaging data.

### 2.3 MRI data acquisition

All visits were performed for research purposes. The exclusion criteria for the participants of the neuroimaging visits were: (1) born before gestational Week 35 (before gestational Week 32 for those with exposure to maternal prenatal synthetic glucocorticoid treatment), (2) developmental anomaly or abnormalities in senses or communication (e.g., blindness, deafness, and congenital heart disease), (3) known long‐term medical diagnosis (e.g., epilepsy and autism), (4) ongoing medical examinations or clinical follow‐up in a hospital (meaning there has been a referral from primary care setting to special health care), (5) child use of continuous, daily medication (including per oral medications, topical creams, and inhalants. One exception to this was desmopressin (®Minirin) medication, which was allowed), (6) history of head trauma (defined as concussion necessitating clinical follow‐up in a health care setting or worse), (7) metallic (golden) ear tubes (to assure good‐quality scans), and routine MRI contraindications.

At the start of the study visit, written informed consent from both parents as well as verbal assent from the child were acquired. Participants were scanned awake or during natural sleep. More detailed visit protocol for age‐appropriate practicalities of the visits is presented in our prior work (Copeland et al., 2021; Pulli et al., 2022). The protocol with incidental findings has been described in our earlier work (Kumpulainen et al., 2020). The image quality assurance followed rigorous procedures combining visual assessments and automated quality checks. For the structural MRI, each T1‐weighted scan was processed with FreeSurfer, manually edited and assessed again after manual edits. We used the ENIGMA consortium quality control protocol to supplement the expert assessments of the 3D images, and provide a full description in Pulli et al. (2022). For the DTI volumes, we first visually assessed the data quality, and then carried out an automated check and exclusion of poor‐quality data using DTIPrep. The automated outlier detection for the DTI data removed images with excessive motion and other artefacts. In the sample, the residual motion in the data was below 0.5 mm for translations (*x*, *y*, *z*) and below 1.3 degrees for rotations around these three principal axes. We also have validated the test–retest reliability (Rosberg et al., 2022) as well as the amount of diffusion encoding directions in our prior work (Kumpulainen et al., 2022).

Participants were scanned using a Siemens Magnetom Skyra fit 3T with a 20‐element head/neck matrix coil. We used Generalized Autocalibrating Partially Parallel Acquisition (GRAPPA) technique to accelerate image acquisition (parallel acquisition technique [PAT] factor of 2 was used). The scanning protocol (maximum length 60 min) included a high‐resolution T1‐weighted Magnetization Prepared Rapid Gradient Echo (MPRAGE), a T2‐weighted Turbo Spin Echo (TSE), a 7‐min resting state functional MRI, and a 96‐direction single shell (*b* = 1000 s/mm^2^) DTI sequence. For the purposes of the current study, we used the high‐resolution T1‐weighted images with the following sequence parameters: repetition time = 1900 ms, echo time = 3.26 ms, inversion time = 900 ms, flip angle = 9 degrees, voxel size = 1.0 × 1.0 × 1.0 mm^3^, field of view 256 × 256 mm^2^. The scans were planned as per recommendations of the FreeSurfer developers (https://surfer.nmr.mgh.harvard.edu/fswiki/FreeSurferWiki?action=AttachFile&do=get&target=FreeSurfer_Suggested_Morphometry_Protocols.pdf, at the time of writing). Also, we used the single shell DTI images with a standard twice‐refocused spin echo–echo‐planar imaging sequence and the following parameters: repetition time/echo time = 9300/87.0 ms, field of view = 208 mm, isotropic voxels with 2.0 × 2.0 × 2.0 mm resolution, *b* value 1000 s/mm^2^, and 96 non‐collinear diffusion gradient directions divided into three scanning sets (31, 32, and 33 directions) with 9 b0 (non‐diffusion‐weighted) images.

### 2.4 Image processing

#### 2.4.1 Structural imaging

The cortical reconstruction and volumetric segmentation for all 173 images were performed with the FreeSurfer software suite (Fischl, 2012), version 6.0.0 (http://surfer.nmr.mgh.harvard.edu/). We selected the T1 image with the least motion artefact (in case there were several attempts due to visible motion during scan) and then applied the ‘recon‐all’ processing stream with default parameters.

After the initial FreeSurfer processing, we visually inspected the images for segmentation errors and manually edited all images. Briefly, the manual edits included removing skull fragments where they affected the pial border, correcting errors in the border between grey and white matter, and removing arteries. After the edits, the FreeSurfer recon‐all was run again. For a more detailed description of the image processing procedure, please see our previous article (Pulli et al., 2022).

The 173 T1 images that passed visual quality control in FreeSurfer pipeline were also included in the voxel‐based morphometry (VBM) analysis. T1‐weighted MRIs were pre‐processed with the Computational Anatomy Toolbox (CAT12; http://www.neuro.uni‐jena.de/cat/, version r1363) for Statistical Parametric Mapping (SPM12; http://www.fil.ion.ucl.ac.uk/spm/software/spm12/, version 7219) software within MATLAB version R2016b. The chosen settings are listed in our prior article (Pulli et al., 2022).

#### 2.4.2 Diffusion tensor imaging

DTIs were pre‐processed as described in detail previously (Rosberg et al., 2022). Briefly, high quality b0 volumes were selected and brain masks created with FSL (FMRIB Software Library, https://fsl.fmrib.ox.ac.uk/fsl/fslwiki/) version 5.0.9 Brain Extraction Tool. Poor‐quality images were removed using DTIPrep (Oguz et al., 2014), and all remaining images were subjected to visual quality control. Motion and eddy currents were corrected using FSL tools, and a diffusion tensor fitted to each voxel in the brain mask using the FSL tool DTIfit ordinary least squares fit. This generated various diffusion scalars, including FA and mean diffusivity (MD).

Images were post‐processed by a modified version of tract‐based spatial statistics (TBSS) in FSL 6.0.7, which involved the use of Advanced Normalization Tools (ANTs; https://github.com/ANTsX/ANTs) to align subject images to the ENIGMA DTI template (https://github.com/pnlbwh/TBSS). With this process, the images were skeletonised by reducing tract diameter of the sample mean image to align with the centre of each tract at a 0.2 FA threshold and projecting each participant's image to the sample mean skeleton. MD images were subsequently projected onto the same FA‐derived skeleton, ensuring both FA and MD values were sampled from identical white matter locations. These skeletonised white matter FA and MD images were used in the current study.

In addition to white matter TBSS analyses, cortical DTI measures were derived by regional averaging of tensor‐derived maps within a cortical parcellation. For each subject, an average b0 image was used as the anatomical reference. Cortical and subcortical segmentations were obtained with FreeSurfer SynthSeg (mri_synthseg) including parcellation output. Voxelwise MD maps were then resampled into the SynthSeg reference space using linear registration. Regional diffusion measures were extracted with FreeSurfer mri_segstats by computing summary statistics within each cortical label (excluding background), using robust estimation and restricting to non‐empty voxels.

#### 2.4.3 Linked independent component analysis of MRIs (FLICA)

**FIGURE 1:**
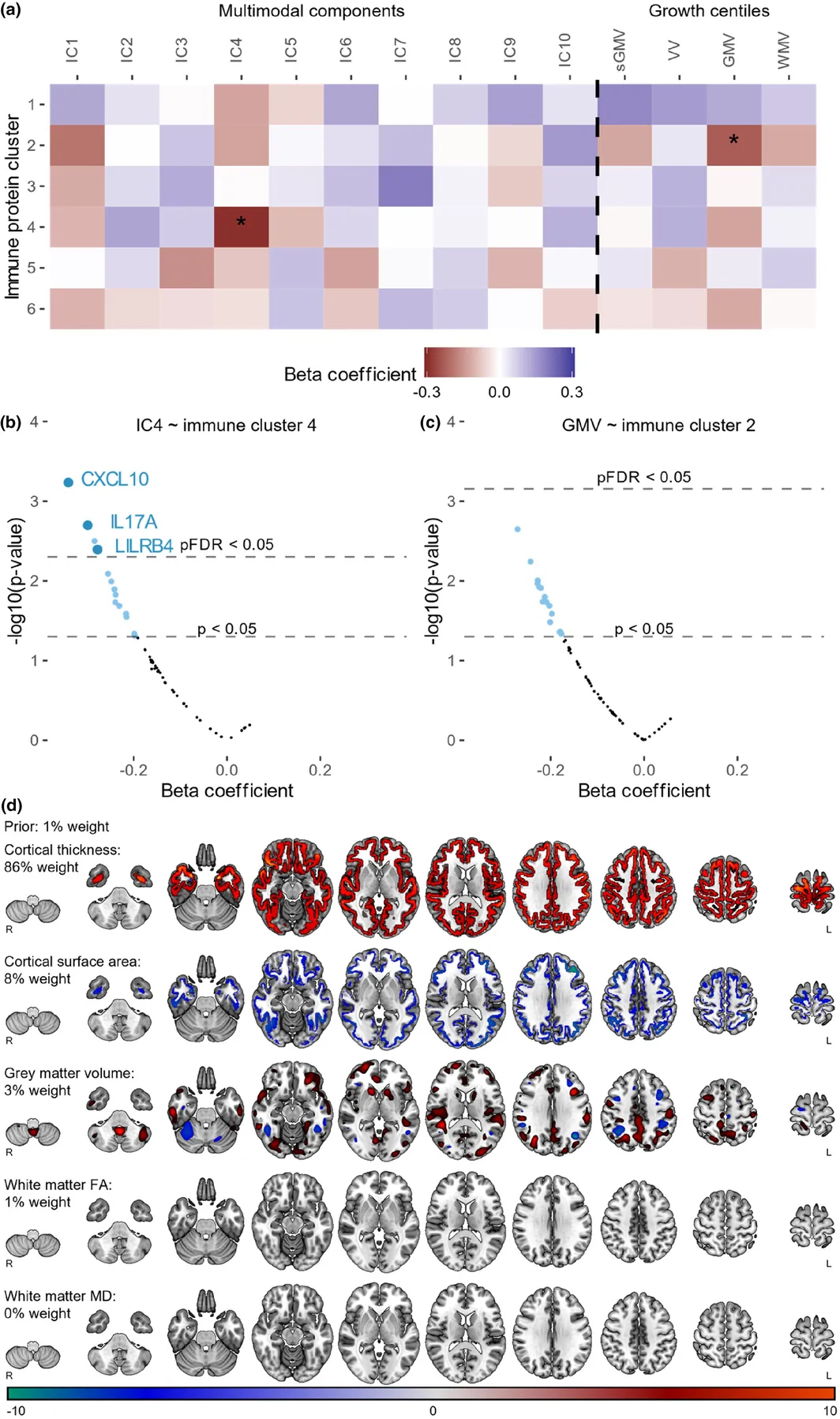
Associations between immune proteome and multimodal brain structural outcomes and volumetric growth centiles. (a) Heatmap of all associations between immune protein clusters and brain structural outcomes. (b) Associations between all individual immune proteins in cluster 4 and independent component 4 (IC4). (c) Associations between all individual immune proteins in cluster 2 and grey matter volume. (d) Anatomical distribution and modality weights of IC4, shown as axial slices in radiological orientation. Statistics represent partial values from linear regression adjusted for sex, age, and ponderal index after FDR adjustment. FA, fractional anisotropy; FDR, false discovery rate; GMV, grey matter volume; IC, independent component; L, left; MD, mean diffusivity; R, right; sGMV, subcortical grey matter volume; VV, ventricular volume; WMV, white matter volume. *p < 0.05 after FDR adjustment.

All pre‐processed MRIs containing distinct voxel‐ or vertex‐wise anatomical information were combined using FSL's linked independent component analysis (FLICA) tools. FLICA links subject images across modalities and uses independent component analysis (ICA) to identify the main multimodal patterns of structural covariance in the data, and this method has been validated both by the original developers (Groves et al., 2011, 2012) and by us in previous work with the current dataset (Barron et al., 2025). Subjects without good‐quality pre‐processed T1‐ and diffusion‐weighted MRI were excluded, and the following images were used as input: cerebral grey matter volume, white matter FA, white matter MD, left and right hemisphere cortical thickness, and left and right hemisphere cortical surface area. FLICA was run with default parameters as per developers' recommendations, which estimated 10 independent components representing the most biological variation across all modalities by performing multiple iterations of ICA until reaching convergence (the point at which the decrease in error of the components between iterations is <0.1) or a maximum of 1000 iterations. The subject‐loading scores constitute a single numeric value per subject per independent component, representing how strongly the subject contributes to that component, and these are the main dependent variables used in the current study. (A manuscript with reports of all 10 components is under review elsewhere, and as such only the one with a significant finding (independent component 4, IC4) is visualised here, with no duplication of figures (Figure 1c)).

### 2.5 Statistics

All statistical analyses were performed using R statistical software version 4.4.0, in RStudio version 2024.09.1 + 394 (https://posit.co/download/rstudio‐desktop/). Immune proteins were clustered by co‐expression using Gaussian mixture modelling with the R package *mclust*, and the clustering algorithm and number of components with the lowest clustering error were chosen, defined by the Bayesian information criterion (BIC).

Associations between immune protein clusters and brain growth centiles, multimodal structural components, or cortical MD were assessed by multiple linear regression adjusting for sex assigned at birth, age at scan, and ponderal index at scan. Multiple hypothesis testing was corrected within each brain measure ~ immune cluster analysis by false discovery rate (FDR) of 5% to obtain *p*‐values corrected for multiple comparisons. In cases where an immune cluster was associated with a given brain structural outcome after FDR adjustment, all individual proteins within that cluster were tested against the respective brain outcome and presented as a volcano plot. Models were run using the base R function lm and partial coefficients and *R* ^2^ values were extracted with the lm.beta and partial_r2 functions from the *lm.beta* and *sensemakr* packages, respectively. All *R* ^2^, beta‐coefficient, and *p*‐values reported in this article represent the partial value for the immune proteins in fully adjusted models with FDR correction. In all cases where multiple immune proteins were examined against a brain structural outcome, the stability of the linear regression coefficients was assessed by bootstrap validation with 1000 iterations of resampling the data with replacement. Beta‐coefficients and *p*‐values from models involving cortical MD were projected onto the cortical surface using the ENIGMA toolbox (https://enigma‐toolbox.readthedocs.io/en/latest/).

## 3 RESULTS

### 3.1 Clustering of immune proteome

The clustering algorithm with the lowest error assigned all 356 inflammation‐associated proteins into one of six clusters, defined as VEI (clusters with variable volume, equal shape, and diagonal orientation, Figure S1A,B). Cluster stability was assessed by repeating the Gaussian mixture modelling with fivefold cross‐validation and estimating the probability that any pair of proteins will be clustered together in the same manner as when clustering was performed on the full dataset. The median probability of identical clustering was 80%. This varied per cluster from 0.67 for cluster 3 proteins to 0.98 for cluster 4 proteins (Figure S1C,D). Although clustering is data‐driven, structurally and functionally related immune proteins are typically grouped together due to their co‐expression. The six clusters comprise mainly (1) interleukin ligands and receptors, and growth factors; (2) immune cell receptors and proteins involved in extracellular matrix remodelling; (3) chemokines and members of the IL‐1 signalling pathway; (4) canonical pro‐inflammatory cytokines such as TNFα, IFNy, IL6, and IL17a, as well as proteins involved in antigen presentation; (5) downstream receptor signalling, mitochondrial activity, redox regulation, and cellular stress response; and (6) NF‐κB and MAPK signaling, hypoxia‐induced transcriptional regulation, and cell adhesion proteins.

### 3.2 Associations between the immune proteome and global metrics of brain development

The final sample size was 101 children with immune proteome and multimodal structural data, and 126 with immune proteome and brain growth centile data. The six immune protein clusters were examined for association with all multimodal brain structural components and growth chart centiles. After multiple testing correction, immune cluster 4 negatively predicted independent component 4 (IC4, *R* ^2^ = 0.09, *β* = −0.29, pFDR = 0.029), while immune cluster 2 negatively predicted grey matter volume growth (*R* ^2^ = 0.05, *β* = −0.23, pFDR = 0.035) (Figure 1a). In both cases, all individual immune proteins in the relevant cluster were then tested against the respective brain outcome. Immune cluster 4 comprises 49 proteins, primarily cytokines and their receptors, chemokines, Fc receptors, and antigen‐presentation proteins. Forty‐four of these (90%) were negatively associated with IC4; 14 of these associations were nominally significant, and three remained significant after FDR adjustment: CXCL10 (*R* ^2^ = 0.15, *β* = −0.33, pFDR = 0.028), IL17A (*R* ^2^ = 0.09, *β* = −0.30, pFDR = 0.049), and SIRPB1 (*R* ^2^ = 0.08, *β* = −0.28, pFDR = 0.049) (Figure 1b). Bootstrap resampling demonstrated that 16 of these proteins had 95% confidence intervals for the bootstrap regression coefficient that did not extend through zero (Figure S2A). Although the immune proteins from cluster 2 exhibit a similar pattern of negative associations with grey matter volume growth, apparent from the highly asymmetrical volcano plot, none of these associations remained significant after FDR adjustment (Figures 1c and S2B). IC4, which was negatively associated with many immune proteins, is composed almost entirely of global cortical thickness (Figure 1d). Therefore, these findings suggest that a cluster of co‐expressed inflammation‐associated proteins, most notably CXCL10, IL17A, and SIRPB1, are negatively associated with indices of cortical development in 5‐year‐old children.

### 3.3 Associations between the immune proteome and cortical microstructure

**FIGURE 2:**
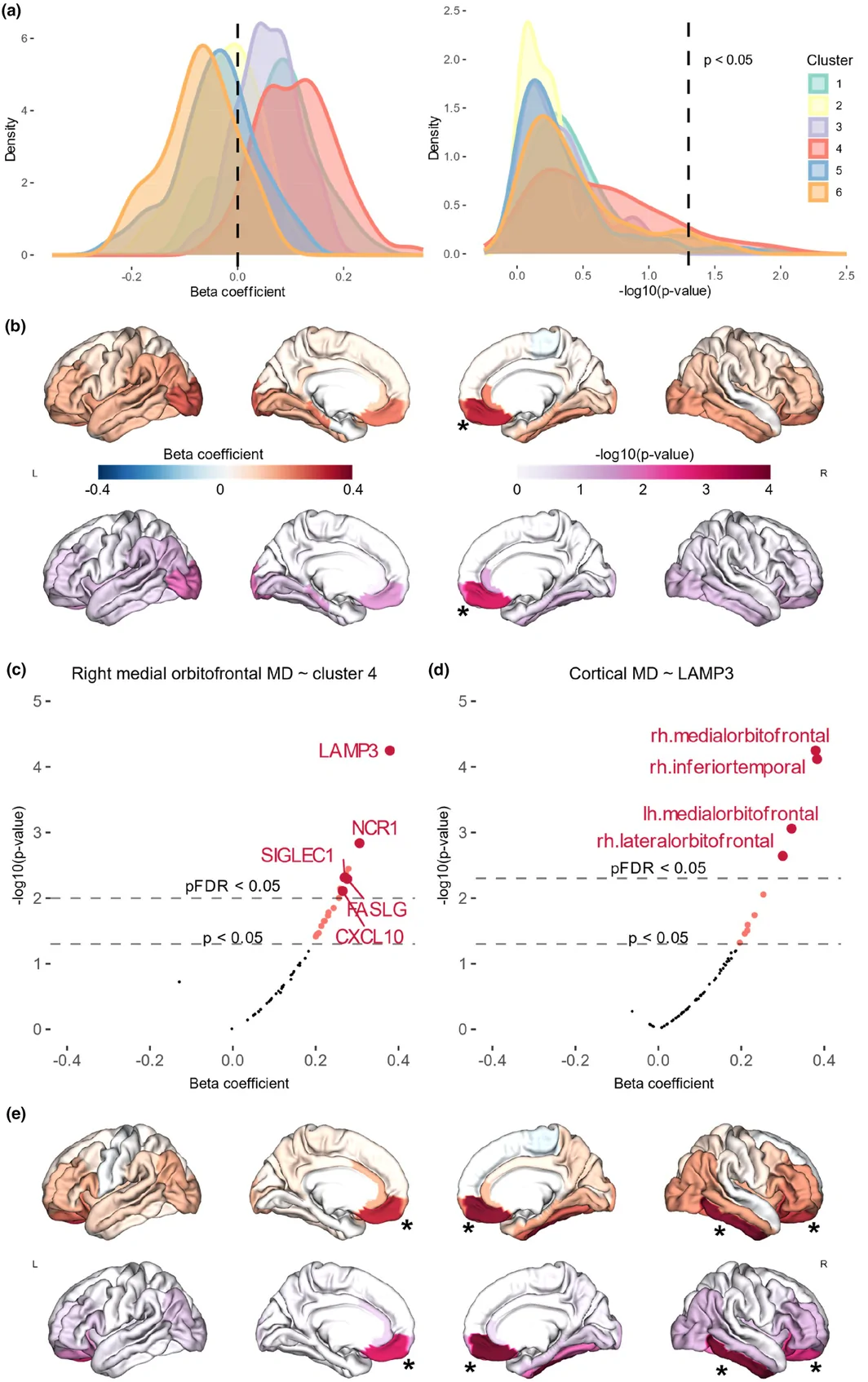
Associations between immune proteome and cortical mean diffusivity. (a) Density plots representing the distribution of effect sizes and *p*‐values from all associations between immune protein clusters and mean diffusivity in cortical regions of interest defined by the Desikan–Killiany (DK) atlas. (b) Anatomical distribution of effect sizes and *p*‐values for association between immune cluster 4 and cortical mean diffusivity. (c) Associations between all individual immune proteins in cluster 4 and mean diffusivity of the right medial orbitofrontal cortex. (d) Associations between LAMP3 and mean diffusivity in cortical regions of interest defined by the DK atlas. (e) Anatomical distribution of effect sizes and *p*‐values for association between LAMP3 and cortical mean diffusivity. Statistics represent partial values from linear regression adjusted for sex, age, and ponderal index after FDR adjustment. FDR, false discovery rate; L, left; MD, mean diffusivity; R, right. *p < 0.05 after FDR adjustment. Common legend for B and E.

We next examined the association between each immune cluster and MD in cortical regions of interest defined by the Desikan–Killiany atlas. Immune cluster 4 exhibited considerably larger effect sizes and lower *p*‐values in these analyses than all other clusters (Figure 2a). Almost all cortical ROIs (64 of 68) have positive associations with immune cluster 4, and after multiple testing correction, this was statistically significant only in the right medial orbitofrontal cortex (*R* ^2^ = 0.11, *β* = 0.32, pFDR = 0.028) (Figure 2b). Next, all individual proteins within this cluster were tested against right medial orbitofrontal MD. A total of 47 of 49 proteins were positively associated with MD; 19 of these were nominally significant, and five significant after FDR adjustment: LAMP3 (*R* ^2^ = 0.15, *β* = 0.38, pFDR = 0.003), NCR1 (*R* ^2^ = 0.10, *β* = 0.31, pFDR = 0.035), FASLG (*R* ^2^ = 0.08, *β* = 0.28, pFDR = 0.041), SIGLEC1 (*R* ^2^ = 0.08, *β* = 0.27, pFDR = 0.047), and CXCL10 (*R* ^2^ = 0.07, *β* = 0.26, pFDR = 0.048), while TNFα had an FDR‐adjusted *p*‐value of exactly 0.050 (Figure 2c). Bootstrap resampling demonstrated that 19 of these proteins had 95% confidence intervals for the bootstrap regression coefficient that did not extend through zero (Supplementary 3).

Lastly, given that LAMP3, a protein involved in dendritic cell antigen presentation, had the notably largest association with MD in this region, its relationship with MD was then explored in all cortical ROIs to determine whether its effects are localised to the right medial orbitofrontal cortex, or more widespread. LAMP3 was positively associated with MD ubiquitously across the cortex, with typically stronger associations in the right hemisphere; these associations were significant after FDR adjustment in the bilateral medial orbitofrontal cortices, and the right inferior temporal and lateral orbitofrontal cortex (Figure 2d,e). Overall, these findings demonstrate that the same family of inflammation‐associated proteins which are associated with gross indices of cortical growth also predict cortical microstructure, with LAMP3 exhibiting the strongest effect.

## 4 DISCUSSION

The present study provides the first characterisation of how early‐life immune protein levels may influence structural brain development in typically developing children. We report robust associations between a family of co‐expressed immune proteins, most notably IL‐17A, CXCL10, and LAMP3, with global cortical neuroanatomical outcomes.

IL‐17A was associated with reduced cortical thickness, consistent with preclinical studies showing that IL‐17A disrupts cortical lamination and induces neurodevelopmental abnormalities following MIA in rodents (Choi et al., 2016; Estes & McAllister, 2016). While no associations with total brain volume were observed, the link between IL‐17A and reduced cortical thickness implicates this cytokine in early cortical organisation in humans. While grey matter volume reflects neuronal changes like neurogenesis, synaptogenesis, and neuronal morphology (Zatorre et al., 2012), postnatal variation in cortical thickness may be more sensitive to individual differences in synaptogenesis, dendritic arborisation and glial modulation (Vidal‐Pineiro et al., 2020). IL‐17A plays a central role in neuroimmune crosstalk, particularly in glial–neuronal interactions and cortical patterning. Thus, the appearance of IL‐17A among the strongest predictors of cortical thickness is strongly in line with previous non‐human literature. Associations between IL‐6 and brain morphology, on the other hand, did not remain significant after correction for multiple comparisons. This was somewhat surprising, given that IL‐6 has been studied extensively in relation to brain development compared with other immune proteins (Graham et al., 2018; Rasmussen et al., 2019; Spann et al., 2018). This discrepancy may be related to the developmental stage: whereas previous studies examined maternal IL‐6 in relation to the brain within the first 2 years, we used immune protein measurements from the child. Additionally, studying a single predetermined immune protein, such as IL‐6, does not introduce the need for multiple comparisons corrections of *p*‐values; in fact, the nominal (unadjusted) *p*‐value for IL‐6 in relation to many brain structural outcomes was indeed significant in this study.

The most widely studied immune proteins may not necessarily be the same as those that are most strongly involved in brain development. With our exploratory approach, we identified immune proteins that have not commonly been studied in this context. C‐X‐C motif chemokine ligand 10 (CXCL10) is a chemokine produced in response to interferon‐gamma (IFN‐γ) activation. Elevated CXCL10 levels have been observed under multiple inflammatory conditions, including infections (Liu et al., 2011) and autoimmune disorders (Rokni et al., 2025). CXCL10 has been implicated in the pathogenesis of multiple sclerosis (Vazirinejad et al., 2014), although its role in typical early‐life brain development is poorly understood. Lysosome‐associated membrane protein 3 (LAMP3) is predominantly expressed in dendritic cells and is involved in antigen presentation. High LAMP3 expression has been associated with chemokine signalling pathways (Wang et al., 2023). Importantly, LAMP3 is mostly expressed in cell types not present in the brain and research related to brain development is very limited. Other immune proteins that were associated with cortical outcomes after multiple testing correction are the immunosuppressant receptor, leukocyte immunoglobulin‐like receptor subfamily B member 4 (LILRB4), mostly expressed on monocytes; the cell adhesion protein sialoadhesin (SIGLEC‐1); natural cytotoxicity triggering receptor 1 (NCR‐1), an activating receptor expressed on the cell membrane of natural killer cells; and Fas ligand (FASLG), which can exist as either a membrane‐bound or secreted ligand and induces apoptosis by binding the Fas receptor. However, it is flawed to think of these proteins as fundamentally different to the others with which they are co‐expressed, given that bootstrap resampling demonstrated that many other immune proteins are significantly associated with the respective brain outcomes. Furthermore, while some of these proteins are expressed in the brain or can cross the blood–brain barrier, others are exclusively peripheral and are therefore better thought of as peripheral biomarkers of immune processes related to brain structure, rather than necessarily causally implicated in brain development. Therefore, these associations do not necessarily indicate a direct neurotropic effect of IL‐17A. The underlying mechanism may involve secondary mediators.

Some limitations to this work should be noted. First, the cross‐sectional design limits causal inference, making it difficult to establish directionality and to speculate on the mechanisms underlying the associations between immune activation and longitudinal growth trajectories. Second, the reliance on peripheral immune protein measures may not fully capture central neuroimmune activity. The moderate sample size of this study compounds this issue, as we may not be able to capture associations with small effect sizes. Also, the method used in this study provides semi‐quantitative, normalised relative expression values rather than concentrations, which limits direct quantitative comparisons with other literature. Comprehensive immune protein assays are technically demanding and less routinely implemented across large‐scale studies, which can constrain sample size, replication, and comparability, and the methodological approach constitutes a major strength of this study. While our results were robust the lack of explicit partial volume correction during diffusion metric extraction represents a limitation when interpreting cortical microstructural measures in young populations. Although immune protein levels in healthy adults are remarkably stable over the course of weeks, months, or even years in the absence of acute injury or infection (Brodin & Davis, 2017), this is less well characterised in 5‐year‐old children. Immune protein levels may therefore be affected by recent infections or medication use, which were not systematically assessed in the present study and therefore represent a limitation. Although participants were drawn from a generally healthy, typically developing population and did not have any acute illness or infection at sample collection, transient physiological factors may nonetheless have influenced immune protein levels and contributed to inter‐individual variability. Future studies with multiple sampling and more detailed clinical characterisation will be important for disentangling developmental and health‐related influences on inflammatory markers.

## 5 CONCLUSIONS

In conclusion, our findings suggest that a group of co‐expressed immune proteins, including IL‐17A, CXCL10, and LAMP3, exhibit convergent associations with early childhood cortical development. These immune proteins were typically associated with reduced cortical thickness and grey matter volume, and correspondingly higher cortical MD, especially in the right medial orbitofrontal region. Although these morphometric features may reflect distinct underlying biological processes, all associations highlight the cortex as a shared target of early‐life immune signaling. This convergence points to specific immune pathways that may shape cortical maturation during a sensitive developmental window. Understanding how individual immune proteins influence different aspects of cortical structure could enhance efforts to identify early biomarkers of neurodevelopmental risk and inform future strategies for targeted intervention.

## AUTHOR CONTRIBUTIONS

**Aaron Barron:** Conceptualised the study, performed FLICA modelling and statistical analyses, wrote the initial draft of the manuscript. **Aylin Rosberg:** Conceptualised the study, wrote the initial draft of the manuscript, extracted cortical DTI metrics. **Jetro J. Tuulari:** Conceptualised the study, funded the data collection for MRI data, planned and led the data collection, supervised the neuroimaging data preprocessing, and FLICA modelling and statistical analyses. **Minna Lukkarinen:** Organised and funded the blood sample collection for the immune proteomics data, planned and led the data collection. **Harri Merisaari:** Processed the neuroimaging data with special input to the DTI data. **Eero Silver:** Collected data, processed the neuroimaging data. **Venla Kumpulainen:** Collected data, processed the neuroimaging data. **Anni Copeland:** Collected data, processed the neuroimaging data. **Ekaterina Saukko:** Collected data. **Alex Dickens** Conceptualised the study, funded the data collection for the immune proteomics data, supervised immune protein analyses. **Matej Oresic** Conceptualised the study, funded the data collection for the immune proteomics data, supervised immune protein analyses. **Tuulia Hyötylainen** Conceptualised the study, funded the data collection for the immune proteomics data, supervised immune protein analyses. **Linnea Karlsson:** Funded the studies, conceptualised and founded the FinnBrain Birth Cohort study. **Hasse Karlsson:** Funded the studies, conceptualised and founded the FinnBrain Birth Cohort study. **Isabella L. C. Mariani Wigley:** Conceptualised the study, wrote the initial draft of the manuscript. **Elmo P. Pulli:** Conceptualised the study, collected data, processed the neuroimaging data, wrote the initial draft of the manuscript.

## FUNDING INFORMATION

JJT: Juho Vainio Foundation; the Hospital District of Southwest Finland, Finnish State Grants for Clinical Research (ERVA); Emil Aaltonen Foundation; Alfred Kordelin Foundation; Sigrid Jusélius Foundation; Signe and Ane Gyllenberg Foundation; the Orion Research Foundation. Open Access funding provided by University of Turku (including Turku University Central Hospital). EPP: Strategic Research Council (SRC) established within the Research Council of Finland (#352648 and subproject #352655), Signe and Ane Gyllenberg Foundation, Finnish Cultural Foundation. AR: Signe and Ane Gyllenberg Foundation. HK: Finnish State Grants for Clinical Research (ERVA), Signe and Ane Gyllenberg Foundation, Research council of Finland (#314390, #332444). LK: Finnish State Grants for Clinical Research (ERVA), Signe and Ane Gyllenberg Foundation. ML: Finnish State Grants for Clinical Research (ERVA).

## CONFLICT OF INTEREST STATEMENT

The authors declare no conflict of interest.

## Supporting information


**Supplementary Figure 1:** Clustering of immune proteome. (A) Guassian mixture modelling was used to cluster 356 inflammation‐association proteins, applying six model types over a range of two 9 clusters. The clustering model and component number combination with the lowest error was chosen to classify the immune proteins and has been circled. (B) Principal components analysis (PCA) bi‐plot demonstrating clustering of immune proteins chosen from (A). Note, the percentage of variance explained shown in axis titles reflects PCA of immune proteins, not participants. (C) Cluster stability analysis involved repeating the Gaussian mixture modelling with fivefold cross‐validation, and estimating the probability that any pair of proteins will be clustered together in the same manner as when clustering was performed on the full dataset. Density plot shows the distribution of these probabilities per cluster. (D) The probability that each individual protein will be clustered with the same proteins in cross‐validated clustering as when the model was fit to the full data. Dashed lines in C and D represent the median probability of 80%. Common legend for C–D, Principal components; BIC, Bayesian information criterion; PC, principal component; VEI, clusters with variable volume, equal shape, and diagonal orientation.
**Supplementary Figure 2:** Bootstrap linear regression for associations between single immune proteins and multimodal brain structural outcomes and volumetric growth centiles. Models were repeated by bootstrapping which resampled the data 1000 times with replacement (A) Associations between all individual immune proteins in cluster 4 and independent component 4 (IC4). (C) Associations between all individual immune proteins in cluster 2 and grey matter volume. Dashed line represents a coefficient of zero. Points and error bars for each immune protein represent the median coefficient and 95% confidence interval across 1000 resamples, respectively.
**Supplementary Figure 3:** Bootstrap linear regression for associations between single immune proteins and cortical mean diffusivity. Models were repeated by bootstrapping which resampled the data 1000 times with replacement Associations between all individual immune proteins in cluster 4 and mean diffusivity of the right medial orbitofrontal cortex. Points and error bars for each immune protein represent the median coefficient and 95% confidence interval across 1000 resamples, respectively.

## Data Availability

The current Finnish legislation and our Ethical Board approval do not permit the open data sharing of the imaging data or derived measures. Investigators interested in getting access to the data are encouraged to contact FinnBrain's Principal Investigators (https://sites.utu.fi/finnbrain/en/contact/). The analysis code can be made available upon a reasonable request to the corresponding author.
