# Supplementary material for: Immune proteomic associations with indices of cortical development in 5‐year‐old children
Source: J Anat. 2026 Sep 10:10.1111/joa.70225. Online ahead of print. doi: 10.1111/joa.70225 (PMC13562773; doi:10.1111/joa.70225)
Supplement: Supplementary file 1 [file JOA-9999-0-s001.zip › Figure S1-S3.docx]

**Supplementary Figure 1: Clustering of immune proteome**. **(A)** Guassian mixture modelling was used to cluster 356 inflammation-association proteins, applying six model types over a range of two nine clusters. The clustering model and component number combination with the lowest error was chosen to classify the immune proteins and has been circled. **(B)** Principal components analysis (PCA) bi-plot demonstrating clustering of immune proteins chosen from (A). Note, the percentage of variance explained shown in axis titles reflects PCA of immune proteins, not participants. **(C)** Cluster stability analysis involved repeating the Gaussian mixture modelling with five-fold cross validation, and estimating the probability that any pair of proteins will be clustered together in the same manner as when clustering was performed on the full dataset. Density plot shows the distribution of these probabilities per cluster. **(D)** The probability that each individual protein will be clustered with the same proteins in cross-validated clustering as when the model was fit to the full data. Dashed lines in C and D represent the median probability of 80%. Common legend for C - D Principal components BIC = Bayesian information criterion, VEI = clusters with variable volume, equal shape, and diagonal orientation, PC = principal component.

**Supplementary Figure 2: Bootstrap linear regression for associations between single immune proteins and multimodal brain structural outcomes and volumetric growth centiles**. Models were repeated by bootstrapping which resampled the data 1000 times with replacement **(A)** Associations between all individual immune proteins in cluster 4 and independent component 4 (IC4). **(C)** Associations between all individual immune proteins in cluster 2 and grey matter volume. Dashed line represents a coefficient of zero. Points and error bars for each immune protein represent the median coefficient and 95% confidence interval across 1000 resamples, respectively.

**Supplementary Figure 3: Bootstrap linear regression for associations between single immune proteins and cortical mean diffusivity**. Models were repeated by bootstrapping which resampled the data 1000 times with replacement Associations between all individual immune proteins in cluster 4 and mean diffusivity of the right medial orbitofrontal cortex. Points and error bars for each immune protein represent the median coefficient and 95% confidence interval across 1000 resamples, respectively.
